# Experimental and numerical investigations on the bending capacity of cold-formed steel box headers

**DOI:** 10.1038/s41598-024-65805-8

**Published:** 2024-07-10

**Authors:** Ahmed A. Matloub, Sara N. Elayouby, Sherif M. Ibrahim, Abdelrahim K. Dessouki

**Affiliations:** Department of Structural Engineering, Ain-Shams University, Cairo, Egypt

**Keywords:** Cold-formed, Built-up box, Experimental program, Header beams, Finite element model, Flexural strength, Civil engineering, Metals and alloys

## Abstract

The study investigates the bending strength of tracks of box headers beyond AISI, which considers the capacity of individual channels alone. Both experimental and FEM are used, and the results are compared to AISI. The findings highlight tracks' significant role in the overall bending capacity. AISI is found to be conservative by 34% to 152%. Failure mode is different from code theoretical expectations for a single channel. Fastener close spacing marginally improves the capacity, while side fasteners offer significant enhancement, but track widening limits this enhancement. A modification to AISI is proposed considering track strength, with outcomes showing good accuracy.

## Introduction

**Figure 1:**
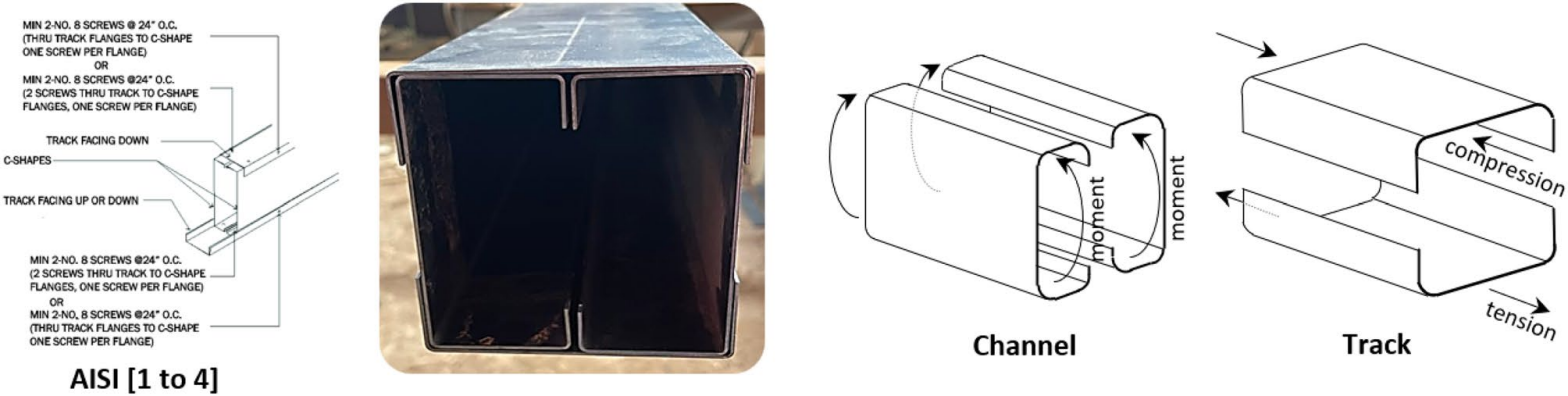
A header built-up box section subject to bending.

Light gauge structures are rapidly increasing nowadays for their advantages. These systems show easy handling, fast construction, and significant economical solutions. This paper assesses the performance of a built-up steel section used in these systems. The section comprises two lipped channels and two tracks arranged to form a box section configuration under bending, as shown in Fig. 1. The closed-profile configuration is arranged by attaching tracks to channels using self-drilling screws. This type of profile is commonly used in light gauge structures, where it serves as header beams spanning large openings in steel-framed bearing walls. The flexural capacity is conventionally limited to the combined strength of the individual channels, with the tracks typically excluded from consideration in adherence to design codes^1–4^. This paper aims to assess and evaluate the role of tracks in the overall flexural capacity of the built-up box section. The contribution of the track is added to the channels’ strength representing the complete capacity of the header beams; consequently, filling the gap in the design codes which account for the bending capacity of channels only. Throughout the discussion, the toe-to-toe stiffened C-channels are referred to as "channels", while the capping U-section (rotated unstiffened C-channel) is referred to as "tracks" for clarity.

C-shaped cold-formed sections are commonly used in steel buildings due to their impressive stiffness-to-weight ratio. However, these sections often have a reduced moment capacity due to different failure modes like local buckling (LB), distortion buckling (DB), and lateral torsional buckling (LTB). A double symmetric shape, which offers higher capacity than individual channels, has been introduced to overcome this limitation. The behavior of built-up profiles has gained significant attention in research, particularly as columns and beams. Built-up sections are presented in the literature as I-shapes formed by back-to-back channels or box-shapes comprised of two overlapping toe-to-toe plain channels. Numerous studies have concluded that built-up sections hold more promise than individual profiles.

As columns, research has highlighted that AISI tends to be conservative in predicting compression capacity, whether under pin-pin or more rigid boundary conditions. Improvement in DB of built-up box sections has been observed, though LB remains a challenge in parts between screws. The slenderness of flat elements influences the compression capacity of built-up columns^5–11^.

As beams, numerous studies have focused on the behavior of built-up sections under bending. Built-up I-shapes exhibit double the capacity of individual channels, while box shapes generally have higher strength^12^. Buckling modes differ between built-up and individual sections, with LTB failure mode recorded for built-up I-shapes and DB for box-shapes, showing considerable resistance^13^. Flange slenderness affects the flexural capacity of built-up I-shapes, while beam span and web slenderness ratio have little influence^14^. In the case of non-symmetry in the outside direction, the total capacity of the two individual sections should be reduced^15^. Additionally, eccentric loading prompts stress unevenness, resulting in lower capacity due to twisting^16,17^. The conservatism of the codes and unreliable prediction of the failure mode for the closed built-up beams is a highlighted issue^18–20^.

There are two methods used to join individual sections into a unified profile. The first approach involves welding the interface parts through resistance spot welding^21^ or laser welding, which offers advantages such as automation, high quality, and minimal heat^22^. However, the welding process to such thin-walled sections shall receive high attention to avoid common problems such as warping or residual stresses^23^. The second method involves mechanical fasteners like self-drilling screws. The spacing between screws influences failure, mainly in compact sections^13^. Meanwhile, screws in the web do not affect the flange's LB, whereas screws in the flanges do^18,19^. For I-shaped sections combined with channel separation, extensive screw spacing can lead to LTB^24^. For built-up back-to-back channels with intermittent links, spacing between links affects flexural capacity, while the gap between channels has a more pronounced effect^25^. Capping this section with a U-profile puts LTB off, causing a bending capacity increase, while increasing spacing between fasteners decreases bending capacity due to LB^26^.

Lastly, web crippling arises due to stress concentration in the case of built-up box shapes under interior-one-flange loading. The combined crippling strength and bending strength exceed the summation of two independent C-sections^27^.

Numerous studies have focused on built-up sections, but the approach in current design codes uses the strength of individual channels alone to calculate the capacity of a built-up section ignoring the tracks. This paper assesses the performance of tracks to account for their strength in the overall capacity of box headers. The analysis includes both experimental investigations and finite element analyses. The study is limited to a box section that comprises channels and tracks. The material is steel of normal grade. The section utilizes architectural walls with a 200 mm width and services an opening with a width of less than 1.8 m. The headers are considered simply supported and laterally unrestrained along their span. Different slenderness ratios of channels and different spacing configurations between screws are covered. Finite-element analysis is performed on the specimens using ABAQUS^28^ to improve the investigation.

## Experimental program

**Figure 2:**
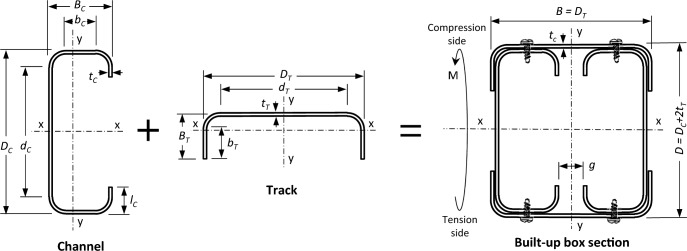
Cross-section of the specimen.

Thirteen specimens are tested in a four-point bending test to have a constant moment at the middle zone of the span. Each specimen consists of two channels with lips positioned toe-to-toe and set in direct contact or with a gap. The channels are encased at their upper and lower flanges using tracks, as shown in Fig. 2. The tracks are fixed to the channels using self-drilling screws. The bending moment is applied to the channels' major axis (x-axis). This experimental program is conducted at the Faculty of Engineering laboratory at Ain Shams University.

The individual sections are cold-formed from two different parent sheets using the press-braking method. The inner radius of all corners is 2.0 mm. Laser marking is used to set the cut lengths and rolling locations, ensuring the accuracy of specimen dimensions. Each specimen is uniformly set to a fabricated length of 2000 mm to serve normal widths of windows and doors, with a beam span of 1800 mm accounting for a 100 mm end distance beyond the support.

**Figure 3:**
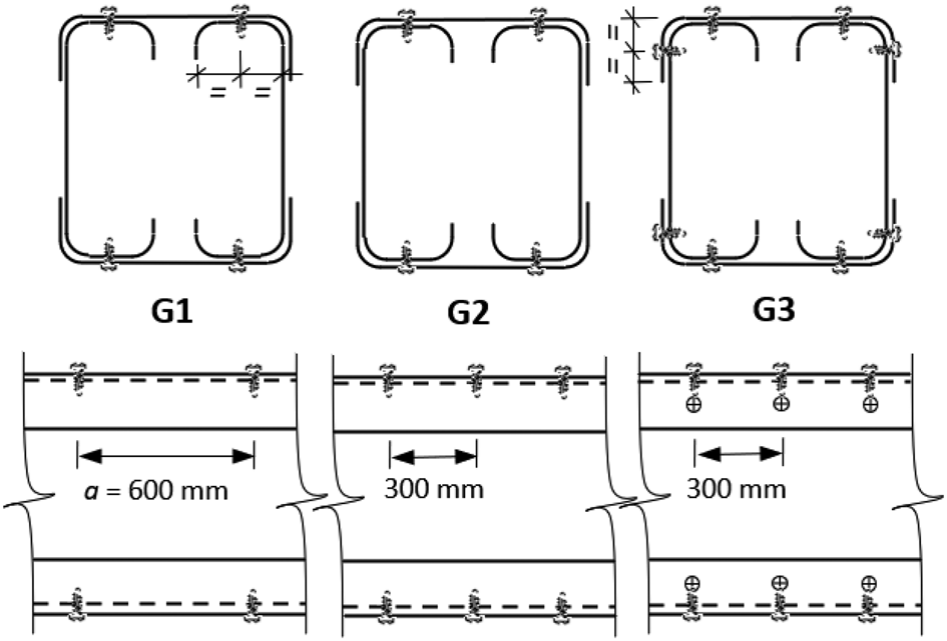
Screws arrangement.

After fabrication, tracks are fastened to the channels using screws measuring 5.0 mm in diameter and 10 mm in length. The screw arrangement follows a pattern, with screws positioned along the midsection of the channels' flanges to the tracks' webs. The original spacing between screws (*a*) along the longitudinal axis is set at 600 mm, adhering to AISI regulations of 24 inches as a maximum. Different screw arrangement is tested to evaluate its effect on the flexural capacity. In certain specimens, this spacing is halved to 300 mm, and in other specimens, additional screws are inserted between the track's flange and the channel's web. The configurations of groups G1, G2, and G3 are illustrated in Fig. 3.

The cross-sections of channels are selected from a list provided in AISI^1^. Three depths of channels (*D*_*C*_) are chosen, which are 92, 150, and 200 mm. Two overall widths of the built-up box section (track depth) (*D*_*T*_) of 92 and 150 mm are chosen. The channel’s flange width (*B*_*C*_) is always 45 mm, and the gap (*g*) between individual channels is 60 mm in three specimens and zero in the others. The channel’s lip depth (*l*_*C*_) is 15 mm, and the track’s flange width (*B*_*T*_) is 30 mm.

Two thicknesses are selected for the channels to achieve different slenderness classifications. The flat width-to-thickness ratio of the channel’s flange (*b*_*C*_/*t*_*C*_) is assessed against the limit 1.12 x (*E*/*F*_*y*_)^0.5 or 1.4 x (*E*/*F*_*y*_)^0.5 for the compact or slender class, respectively, since the top flanges are subject to uniform compressive stresses from the bending moment. Meanwhile, the channel’s web is primarily subjected to gradient stresses, so its flat width-to-thickness ratio (*d*_*C*_/*t*_*C*_) is assessed against the limit 3.76 x (*E*/*F*_*y*_)^0.5 or 5.7 x (*E*/*F*_*y*_)^0.5. For the tracks, the one on top is classified as slender due to the large web flat depth-to-track thickness (*d*_*T*_/*t*_*T*_) because it is subject to uniform compressive stresses from the bending moment.

Each specimen is designated with a unique serial number in millimeters, followed by its geometry screw arrangement, to make it easier to present the data. For example, a specimen is labeled as (*D*_*C*_ x *D*_*T*_—*t*_*C*_/*t*_*T*_—*g*—G1) presenting the depth and width of the box section, the thickness of the channel and track, the gap between channels, and the arrangement of the screws, respectively. At the same time, a serial number is given to each specimen as an alternative short naming. The nominal dimensions and the designations for all specimens are listed in Table 1.

**Table 1:** Nominal dimensions of specimens.

Specimen		*D*_*C*_	*B*_*C*_	*D*_*T*_	*t*_*C*_	*t*_*T*_	*g*	*a*
No	Designation	(mm)	(mm)	(mm)	(mm)	(mm)	(mm)	(mm)
S01	92 × 92-1/1-0-G1	92	45	92	1.0	1.0	0	600
S02	92 × 92-1/1-0-G2	92	45	92	1.0	1.0	0	300
S03	92 × 92-1/2-0-G1	92	45	93.7	1.0	1.85	0	600
S04	92 × 92-1/2-0-G2	92	45	93.7	1.0	1.85	0	300
S05	92 × 92-2/1-0-G1	92	45	92	1.85	1.0	0	600
S06	92 × 92-2/2-0-G1	92	45	93.7	1.85	1.85	0	600
S07	200 × 92-1/2-0-G1	200	45	93.7	1.0	1.85	0	600
S08	200 × 92-1/2-0-G3	200	45	93.7	1.0	1.85	0	300^W^
S09	200 × 92-2/1-0-G3	200	45	92	1.85	1.0	0	300^W^
S10	200 × 92-2/2-0-G3	200	45	93.7	1.85	1.85	0	300^W^
S11	150 × 150-1/1-60-G1	150	45	152	1.0	1.0	60	600
S12	92 × 150-1/1-60-G1	92	45	152	1.0	1.0	60	600
S13	92 × 150-2/1-60-G1	92	45	152	1.85	1.0	60	600

^W^Indicates a line of side screws between the track’s flange and the channel’s web.

**Figure 4:**
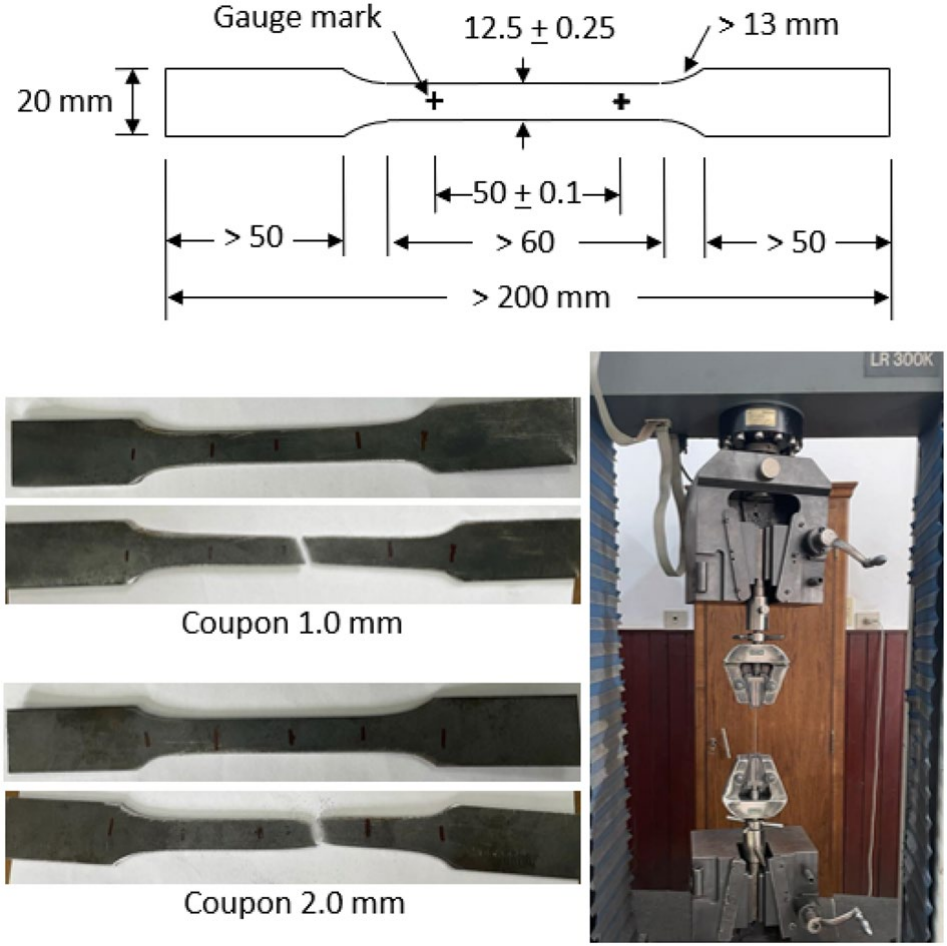
Coupons test.

**Figure 5:**
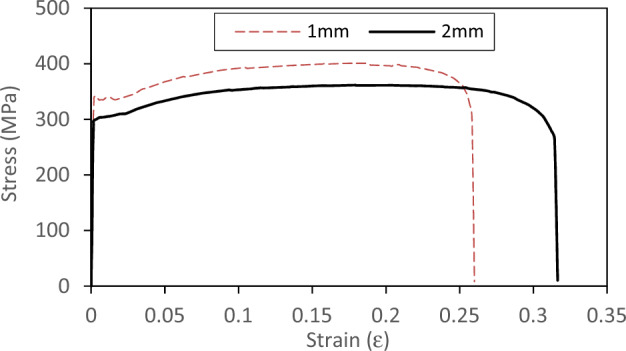
Stress–strain curves from coupon tests.

Tension tests are conducted to determine the mechanical properties of the material. The coupons are taken from the original sheet before the cold rolling process. These coupons are cut from the parent sheet in the longitudinal direction of the beam. The dimensions of the coupons including the gauge length and width, in addition to photos of the machine and coupons before and after the test are presented in Fig. 4. The results for yield stress (*F*_*y*_), ultimate strength (*F*_*u*_), and ultimate strain at fracture (ε_*u*_) for each thickness are compiled in Table 2. The engineering stress–strain curves from the tests are presented in Fig. 5. The modulus of elasticity of steel (*E*) is considered 200,000 MPa up to the yield plateau.

**Table 2:** Average results of coupon tests.

Coupon	*t*	*F*_*y*_	*F*_*u*_	*ε*_*u*_
	(mm)	(MPa)	(MPa)	(mm/mm)
1 mm	1.0	340	401	0.26
2 mm	1.85	297	361	0.316

**Figure 6:**
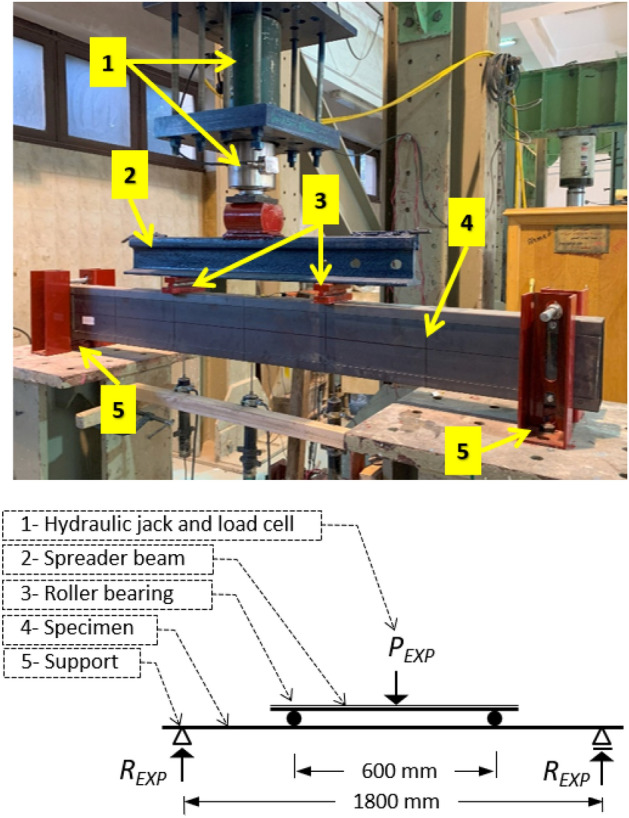
Test setup.

**Figure 7:**
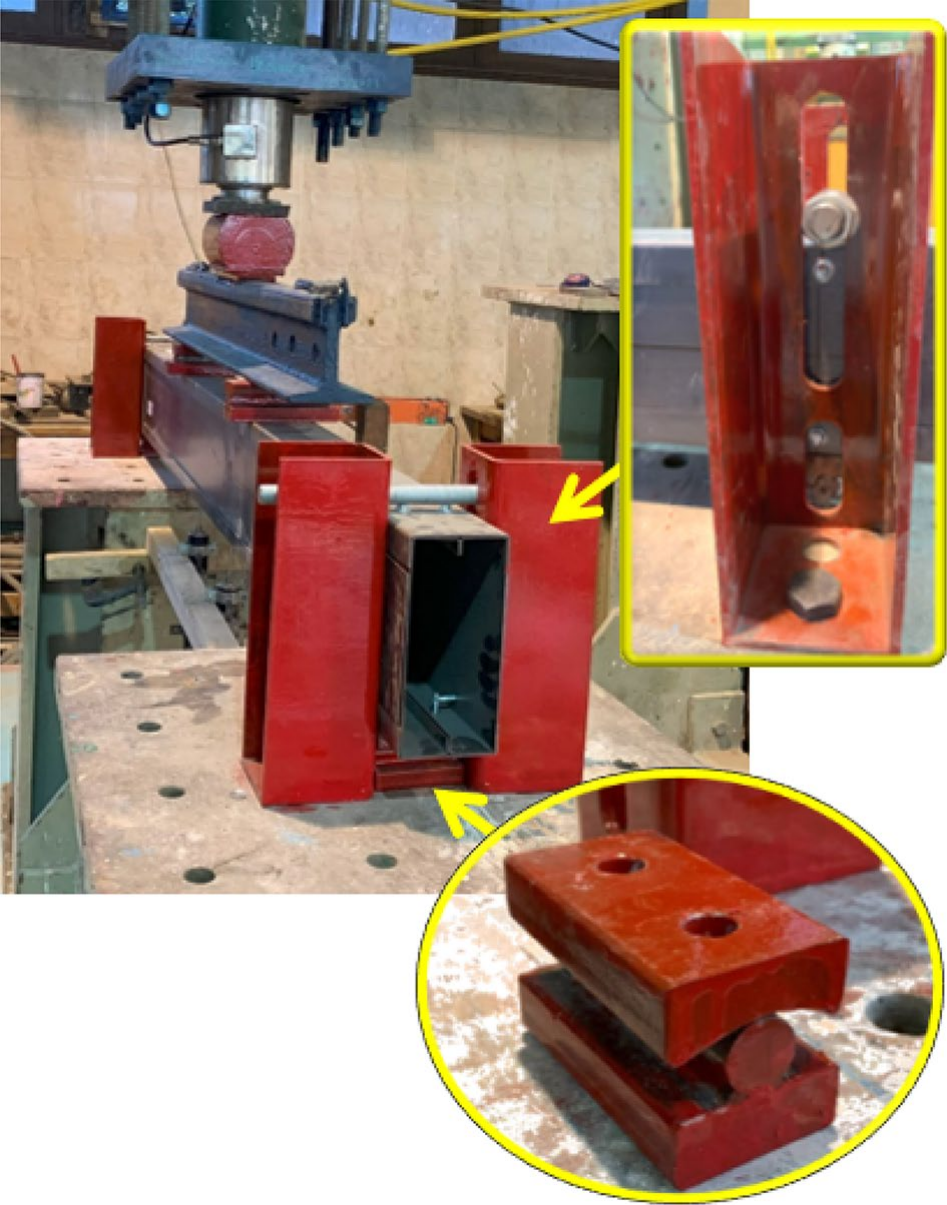
Specimen support.

The four-point bending setup of tests is shown in Fig. 6. The loading process involves applying load incrementally at a controlled rate until failure occurs, followed by unloading. A hydraulic jack applies a concentrated load (*P*_*EXP*_) onto the specimen. The jack is suspended from the laboratory fixed frame and connected to a load cell at the bottom. A rigid spreader beam is used to evenly distribute the load into two points along the span of the specimen. The two points are located at one-third and two-thirds from the supports. At these points, two bearing plates with a thickness of 20 mm, the specimen's width, and a length of 50 mm are placed to prevent local failure. A rod is positioned between the plates to allow free rotation. The same technique is applied to the supports to create a simple supported condition. U-shaped posts are placed adjacent to the specimen at both ends to restrict lateral movement, connected by a rod above it to allow rotation and prevent upward displacement. Oversize grooves are provided in the bearing plates to accommodate the screw heads that are located at supports and load points, ensuring sufficient clearance to prevent any restriction in translation or rotation at the four load points. It is important to note that no web thickening or stiffener additions are introduced under the loads or at the supports to represent real scenarios in the construction of light buildings. Typically, header beams undergo minimal or no treatment at the loaded points. Refer to Fig. 7 for the specimen support configuration.

Three linear variable displacement transducers (LVDTs) are placed beneath the box section to measure the vertical deflection of the beam. They are positioned at each loaded point and the midspan. In addition, two horizontal LVDTs are placed at the midspan to measure torsional rotation. These horizontal LVDTs detect variations in the horizontal displacement between the top and bottom levels.

**Figure 8:**
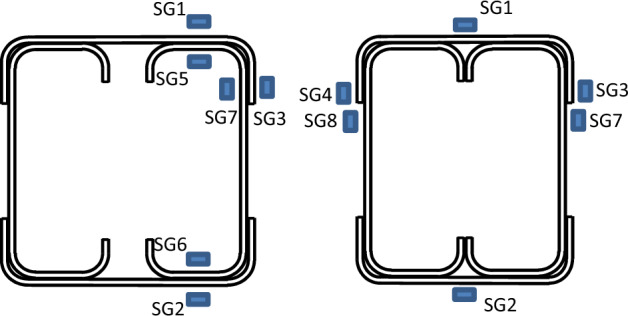
Arrangement of strain gauges.

The strains along the length of a specimen are monitored using strain gauges that are pasted to the midspan of the specimen. Most of the strain gauges are positioned on the tracks, placed at tension and compression sides, along with the flanges of the compressive track. In addition, a strain gauge is fixed to the exposed portion of the channels, precisely beneath the compressive part, just below the track. For specimens that have gaps between channels, three strain gauges are placed at the inner side of one channel before screw drilling. After drilling, three more strain gauges are placed at the same positions but on the tracks. The arrangement of the strain gauges is shown in Fig. 8. All readings of LVDTs, strain gauges, and the load cell are automatically recorded using a data acquisition system.

## Experimental results

**Figure 9:**
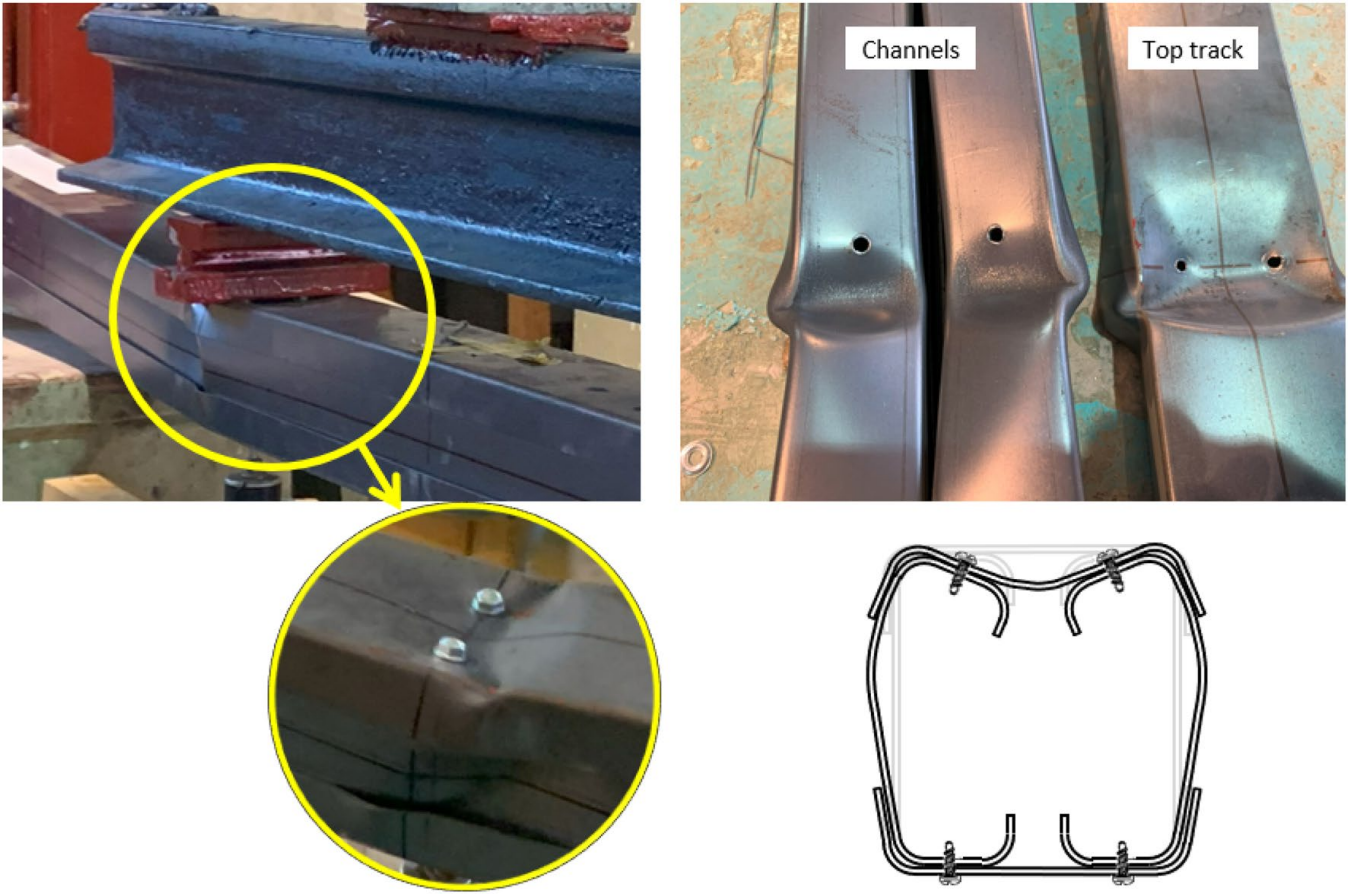
Failure mode.

This section presents the results of the conducted experiments. According to Fig. 9, failure consistently occurred beneath the loaded points. Initially, the top flange of the channel experienced distortion, followed by outward side deformation of the web at the compression side. This distortion of the channel top flange subsequently caused a downward movement of the channel lip, creating a small gap with the bearing plate. As a result, the load became concentrated on the channel web rather than being distributed uniformly across the top surface. With increasing the load, downward LB occurred under the load at the channels’ top flange and the track’s top web. No LTB is observed in any specimen. The top and bottom lateral displacement at the midspan of all specimens record minimal values, tending toward zero.

Table 3 presents the maximum experimental load, *P*_*EXP*_, of each specimen measured by the load cell. The reaction at the support, *R*_*EXP*_, which equals half the experimental load is also included. In addition, the table contains *R*_*y*_, the reaction that causes the yielding moment of the channels only. The yielding moment is calculated by multiplying the elastic section modulus of the two channels by their yielding stress. The results show that the experimental loads exceed the yield of the channels for most specimens, especially for thicker tracks. Load beyond yielding highlights the significant influence of track dimensions on the structural performance of built-up box sections. Later in the paper, there will be further elaboration and analysis of these findings, including comparisons with relevant code specifications.

**Table 3:** Results of experimental tests.

Specimen		*P*_*EXP*_	*R*_*EXP*_* (0.5P*_*EXP*_*)*	*R*_*y*_	*R*_*EXP*_* /R*_*y*_
No	Designation	(kN)	(kN)	(kN)	
S01	92 × 92-1/1-0-G1	15.44	7.72	6.87	1.12
S02	92 × 92-1/1-0-G2	15.96	7.98	6.87	1.16
S03	92 × 92-1/2-0-G1	18.37	9.185	6.87	1.34
S04	92 × 92-1/2-0-G2	19.36	9.68	6.87	1.41
S05	92 × 92-2/1-0-G1	26.39	13.195	10.64	1.24
S06	92 × 92-2/2-0-G1	31.25	15.625	10.64	1.47
S07	200 × 92-1/2-0-G1	36.88	18.44	19.63	0.94
S08	200 × 92-1/2-0-G3	43.05	21.525	19.63	1.10
S09	200 × 92-2/1-0-G3	61.01	30.505	30.85	0.99
S10	200 × 92-2/2-0-G3	71.50	35.75	30.85	1.16
S11	150 × 150-1/1-60-G1	24.14	12.07	13.17	0.92
S12	92 × 150-1/1-60-G1	14.37	7.185	6.87	1.05
S13	92 × 150-2/1-60-G1	26.59	13.295	10.64	1.25
	Average				1.165

**Figure 10:**
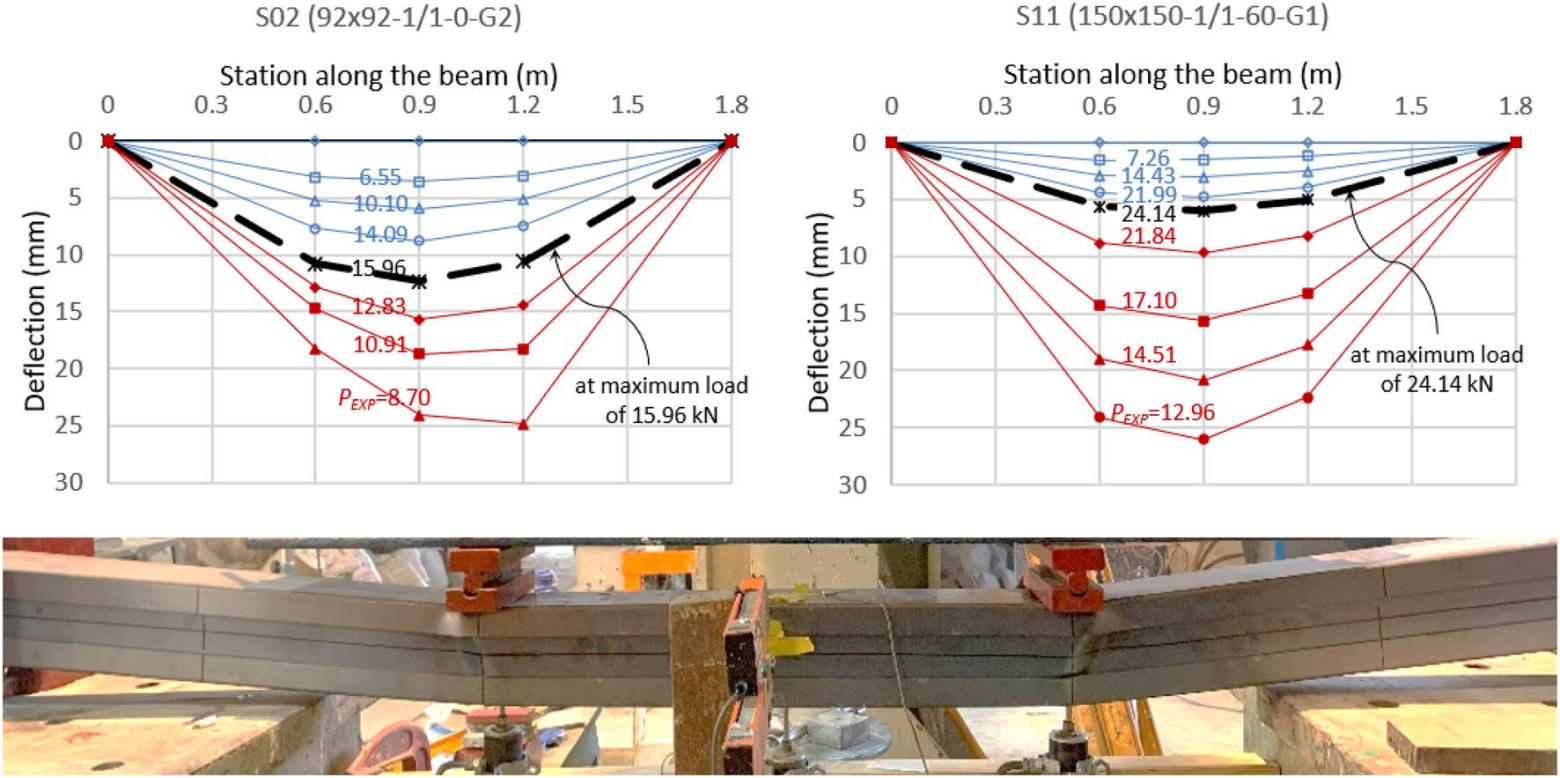
Vertical deflection along specimens.

Figure 10 shows the deflection growth along the span for two samples, S02 and S11, accompanied by a photograph that demonstrates the failure of one of the specimens. Before reaching the ultimate load, the maximum deflection consistently occurs at the midspan of the beam (at station 0.9 m on the horizontal axis). Moreover, the deflection under the two loaded points (at stations 0.6 m and 1.2 m) is almost identical. However, unsymmetrical deflection beyond the maximum load and during the plastic stage is detected for some specimens. Excessive plastic deflection progresses under one loaded point more than the other, as seen in specimen S02 (92 × 92-1/1-0-G2).

**Figure 11:**
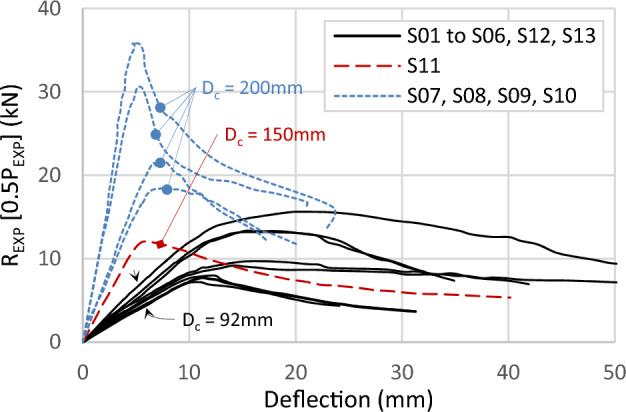
Load–deflection curve for all experimental tests.

Figure 11 compiles the results of all specimens in one graph. The curves in the graph are plotted in terms of the reaction (*R*_*EXP*_ = 0.5*P*_*EXP*_) versus the deflection at the midspan. The ultimate reactions range from 9.0 kN to 35 kN, corresponding to a moment capacity between 5.4 kN.m and 21 kN.m. The elastic deflection at the ultimate load ranges from 5.0 to 20 mm, with a smaller value for the greater depths of the channels. When the depth is 200 mm, the load decreases rapidly after reaching its peak, steeper than the 92 mm depth.

### Thickness of channels and tracks

**Figure 12:**
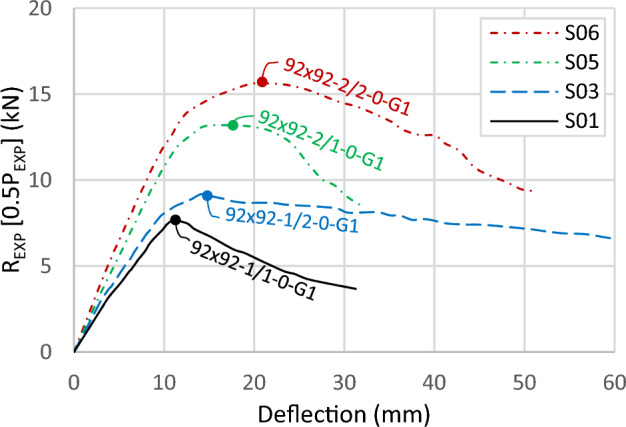
Load–deflection curve for specimens with different channel and track thickness.

Comparative analyses have been undertaken on specific specimens to explore the influence of various parameters. Changing the thickness of sections highlights a significant enhancement in capacity and initial stiffness with increasing channel thickness. An enhancement is also observed with an increase in track thickness, indicating its contribution to the capacity of the built-up section. As shown in Fig. 12, specimen S05 (92 × 92-2/1-0-G1) exhibits a 71% higher capacity than S01 (92 × 92-1/1-0-G1), attributing this to the thicker channel. The increase in the channel thickness enhances the initial stiffness and capacity of the built-up section. Similarly, specimens having thicker tracks, such as S06 (92 × 92x2/2-0-G1) and S03 (92 × 92-1/2-0-G1), show a 61% capacity increase, a lesser rise due to the reduced percentage of channel area in the built-up sections with thicker tracks.

The findings highlight the collaborative role of channels and tracks in the total capacity, with the tracks exhibiting notable influence. Figure 12 further demonstrates that increasing track thickness from 1.0 mm to 1.85 mm results in a 19% enhancement in the built-up section's capacity, as evidenced by specimens S05 (92 × 92-2/1-0-G1) and S06 (92 × 92-2/2-0-G1), as well as between S01 (92 × 92-1/1-0-G1) and S03 (92 × 92-1/2-0-G1). Moreover, this increase in track thickness contributes to a stiffer elastic stage and a more ductile behavior after the ultimate load.

**Figure 13:**
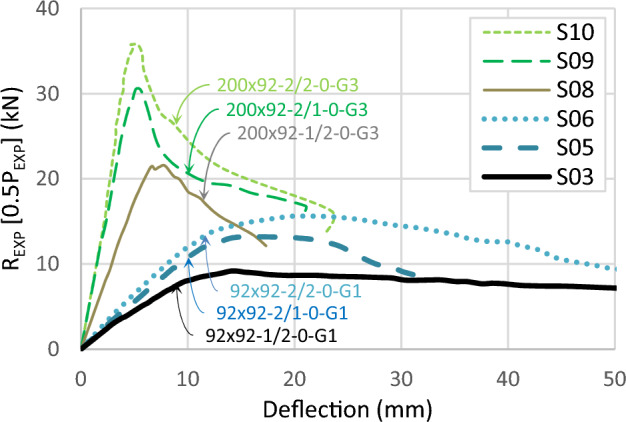
Load–deflection curve for specimens with depth 200 mm and 92 mm.

These trends are consistent across specimens with a depth of 200 mm, as illustrated in Fig. 13. Increasing channel thickness between specimens S08 (200 × 92-1/2-0-G3) and S10 (200 × 92-2/2-0-G3) leads to a 66% capacity increase. Similarly, upgrading track thickness, as seen in specimens S09 (200 × 92-2/1-0-G3) and S10 (200 × 92x2/2-0-G3), results in a 17% capacity increase for the same channel thickness.

**Figure 14:**
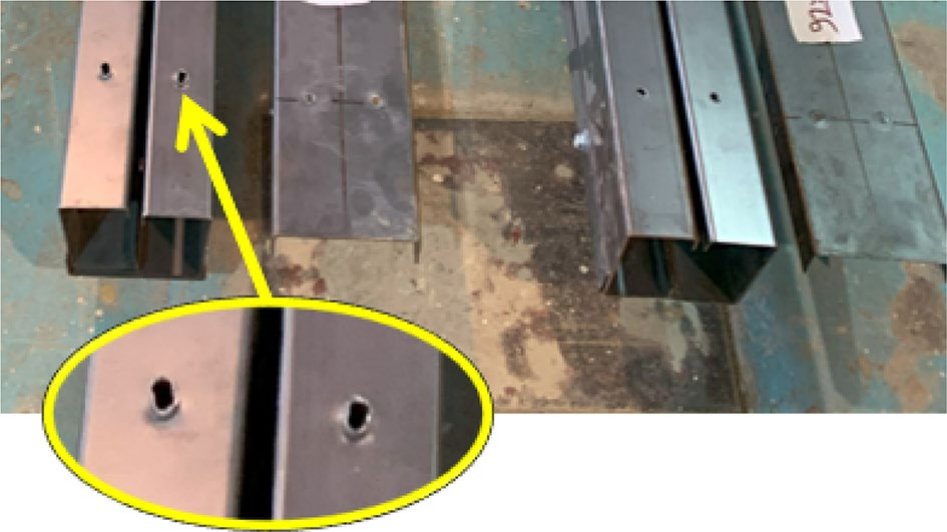
Bearing at channel’s flange.

In summary, tracks play a substantial role in the moment capacity of built-up box sections, with their contribution growing as track thickness increases. The contribution of tracks presents an opportunity for design optimization and increasing section spans. However, caution is needed when the track exceeds the channel in thickness, as illustrated in Fig. 14. Deformations due to screw bearing on a slender channel at the support are evident in specimen S03 (92 × 45 × 1/2-0-G1), where the thick track sustains a large force, resulting in high shear flow at the location of maximum shear. This deformation was unique to one specimen without detecting the same in other specimens with a similar thicker track than channel configuration.

### Gap between channels

**Figure 15:**
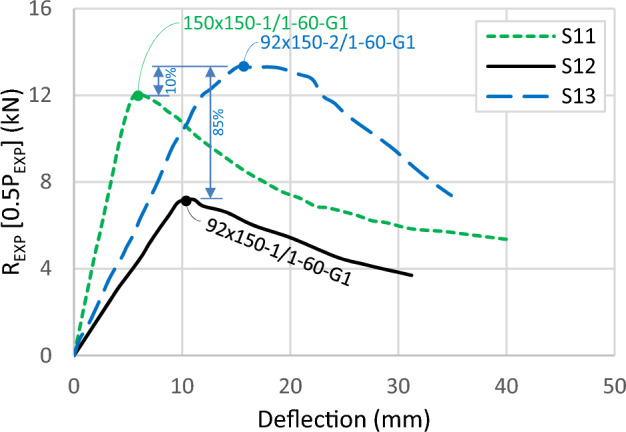
Load–deflection curve for specimens with different channel depths.

Figure 15 illustrates the load–deflection relationship of three specimens, focusing on a second parameter: widening the track and providing a gap between channels. Comparing specimen S13 (92 × 150-2/1-60-G1) with S11 (150 × 150-1/1-60-G1), a 10% higher ultimate load is observed in the shallower section despite both specimens having equal section modulus. The shallower section exhibits higher capacity, while the deeper section demonstrates high initial stiffness. The 150 mm channel's depth with a 1.0 mm thickness is slender, while the 92 mm web with a 1.85 mm thickness satisfies the compact section limit, resulting in the larger capacity of the 92 mm section.

**Figure 16:**
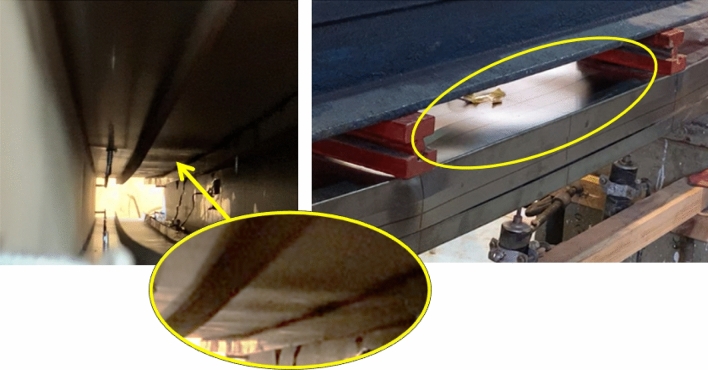
Local buckling in track.

For the third specimen S12 (92 × 150-1/1-60-G1), the small ultimate load and the low initial stiffness are observed. Compared to the same section with a thicker channel, the capacity increases by 85%. This enhancement is higher than the previously detected 71% enhancement in the last paragraph for the same section without a gap. Introducing a gap widens the track and makes it slenderer, increasing the likelihood of LB in the track's web. The slenderer condition limits the sharing of the track and magnifies the channel's contribution, resulting in the recorded maximum enhancement in capacity of 85%. Figure 16 illustrates this result for a model with a track of 150 mm width and 1.0 mm thickness, showing LB waves occurring in the track's web along the length between the loaded points. These elastic deformations were observed during loading and disappeared after releasing the load.

**Figure 17:**
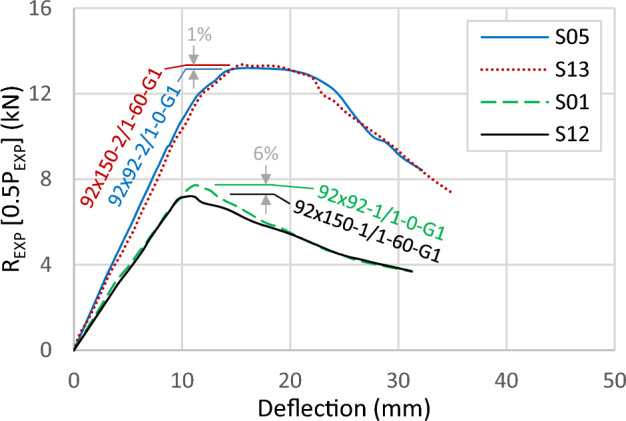
Load–deflection curve for specimens with different track widths.

Figure 17 displays two curves with 0 and 60 mm gaps, showing minor differences despite the increase in the cross-sectional area of the tracks. By increasing the track width, and initiating a gap between channels, the capacity decreases by 6% between specimen S01 (92 × 92x1/1-0-G1) and S12 (92 × 150-1/1-60-G1). The difference in capacity becomes very minor with only 1% between specimen S05 (92 × 92-2/1-0-G1) and S13 (92 × 150-2/1-60-G1). In conclusion, the most significant benefit of the track likely comes from its cross-sectional area, conditioned by keeping the slenderness ratio of the track's web low and retaining the channels' lip in contact.

### Screws arrangement

A proposal to magnify the impact of the track by increasing the number of screws is studied. According to AISI standards, a spacing of 610 mm (24 inches) between screws is insufficient to fully develop composite action between different components of the built-up box section. Consequently, the design of the built-up sections relies solely on the capacity of the channels, excluding the tracks. However, according to the previous results, the influence of the track is significant and should be considered in calculating the built-up section capacity.

**Figure 18:**
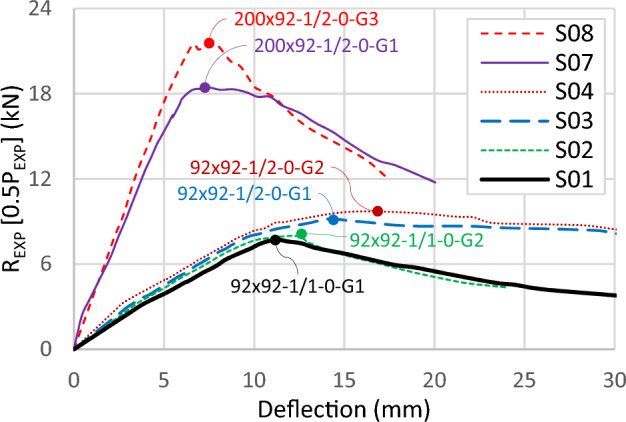
Load–deflection curve for specimens with different screw arrangements.

The study evaluates the impact of screw spacing, focusing on a spacing of 600 mm. Further investigations explore the potential strength gains achievable with smaller spacings between fasteners. Figure 18 illustrates the load-deflection relationship of six specimens grouped into three sets. Specimens with a depth of 92 mm have screw spacings of 600 mm (G1) and 300 mm (G2), while specimens with a depth of 200 mm have screw spacing of 300 mm in addition to adding horizontal screws (G3). The curves within each set exhibit similar trends with minor discrepancies. Specimen S02 (92 × 92-1/1-0-G2) demonstrates a marginal enhancement in ultimate load, approximately 3% higher than specimen S01 (92 × 92-1/1-0-G1). Also, increasing the track thickness shows comparable results, with specimen S04 (92 × 92-1/2-0-G2) exhibiting a 5% higher capacity than specimen S03 (92 × 92-1/2-0-G1). The usage of horizontal screws in specimen S08 (200 × 92-1/2-0-G3) led to a substantial capacity increase of 17%. Across all sets, denser screw arrangements show slightly higher initial stiffness.

In summary, while close fasteners marginally enhance capacity, it is advisable to include additional screws in the channels' web for deeper sections to improve the behavior. Moreover, dense fasteners are recommended for sections with thicker tracks than channels to mitigate high bearing stresses resulting from screws on the thin channel.

### Sharing percentage of track section

**Figure 19:**
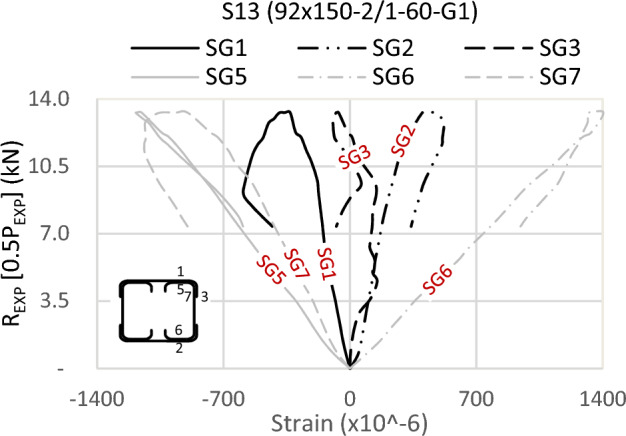
Strains of specimen S13 (92 × 150-2/1-60-G1).

**Figure 20:**
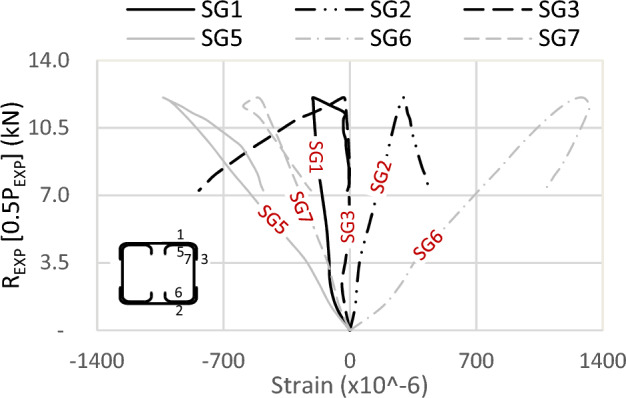
Strains of specimen S11 (150 × 150-1/1-60-G1).

One of the crucial findings from the experimental tests relates to the strain gauge readings. Figures 19 and 20 illustrate the strains in specimens S13 (92 × 150-2/1-60-G1) and S11 (150 × 150-1/1-60-G1), respectively, where strain gauges are pasted at the same location, with one on the track and another on the channel. The maximum strain is observed on the flange of the channels, exhibiting compression at the top (SG5) and tension at the bottom (SG6), with comparable magnitudes. Similarly, the strains in the track section (SG1 at the top and SG2 at the bottom) follow this trend; however, the strain value in the track is 25% of that in the channel. Although the track section significantly contributes to the moment capacity of the built-up section, the composite action is only partial.

Regarding the strain in the channel’s web (SG7), compressive strains are recorded, with values lower than those in the flange. The reason is the closeness to the center of gravity (C.G.) of the channel. As for the strain in the track’s flange (SG3), its behavior is somewhat erratic, lacking a consistent pattern. Initially exhibiting slight compression, it quickly transitions to tension or becomes null. The tip of the top track’s flange is a free edge, indicating an unstressed condition with no contribution to the bending moment of the built-up section.

**Figure 21:**
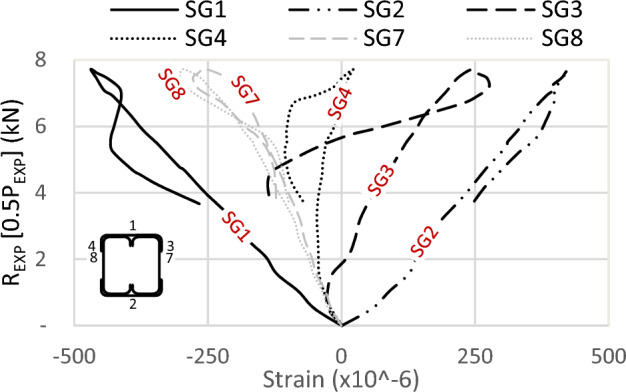
Strains of specimen S01 (92 × 92-1/1-0-G1).

**Figure 22:**
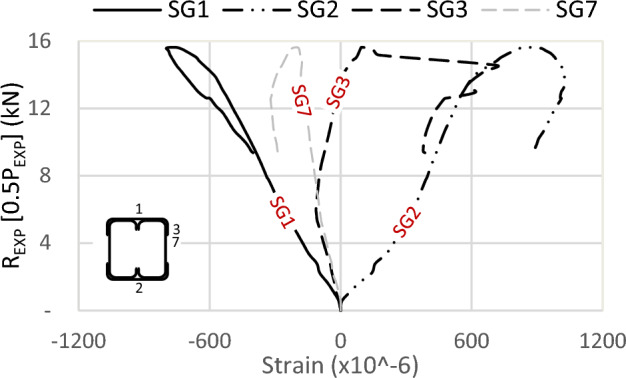
Strains of specimen S06 (92 × 92-2/2-0-G1).

**Figure 23:**
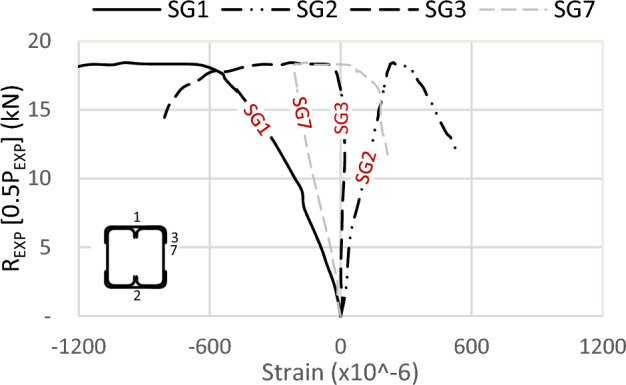
Strains of specimen S07 (200 × 92x1/2-0-G1).

Further strain results are shown in Figs. 21, 22 and 23 for specimens S01 (92 × 92-1/1-0-G1), S06 (92 × 92-2/2-0-G1), and S07 (200 × 92-1/2-0-G1). Since no strains are used at the channels’ flange of these specimens, the maximum strain is recorded on the track’s far fiber, exhibiting compression at the top (SG1) and tension at the bottom (SG2). For the strain in the channel’s web (SG7) and (SG8), compressive strains are measured with identical values on both channels, confirming the absence of LTB or out-of-plane deformation.

Assuming a linear relationship along the section depth, and with the strain gauge on the channel’s web of sections 92 mm located near the C.G. by 10 mm, it is proposed that the strain on the channel’s flange (46 mm above C.G.) is 4.6 times the recorded strains by SG7. This assumption results in strains in the channel’s flange two times the recorded strains SG1 in the track in Fig. 21 and 1.6 times in Fig. 22. This ratio translates to partial composite action of 50% and 62% in specimens S01 (92 × 92-1/1-0-G1) and S06 (92 × 92-2/2-0-G1), respectively. By the same calculation, and reference to specimen S07 (200 × 92-1/2-0-G1) in Fig. 23, the percentage of composite action becomes 35%. Regarding the strain in the flange of the track (SG3) and (SG4), the performance again appears arbitrary.

In conclusion, the track section adds 50% of the channel capacity. The influence of the track increases as the track section becomes thick. However, this contribution diminishes to 25% when the track becomes slenderer. Additionally, the track contribution decreases with an increase in the depth of the channels, but this can be mitigated by adding horizontal screws to the channel web.

## Verification using FEM and design code

Several methods can be used to determine the strength of steel members along with laboratory tests to cover a wide range of parameters. One of these methods is machine learning which has been employed in different structural applications^29–31^. Another method is the finite element analysis, an important research pillar^32–34^. Computer software based on the finite element method is now widely used practically in all branches of engineering for the analysis of structures, solids, and fluids.

**Figure 24:**
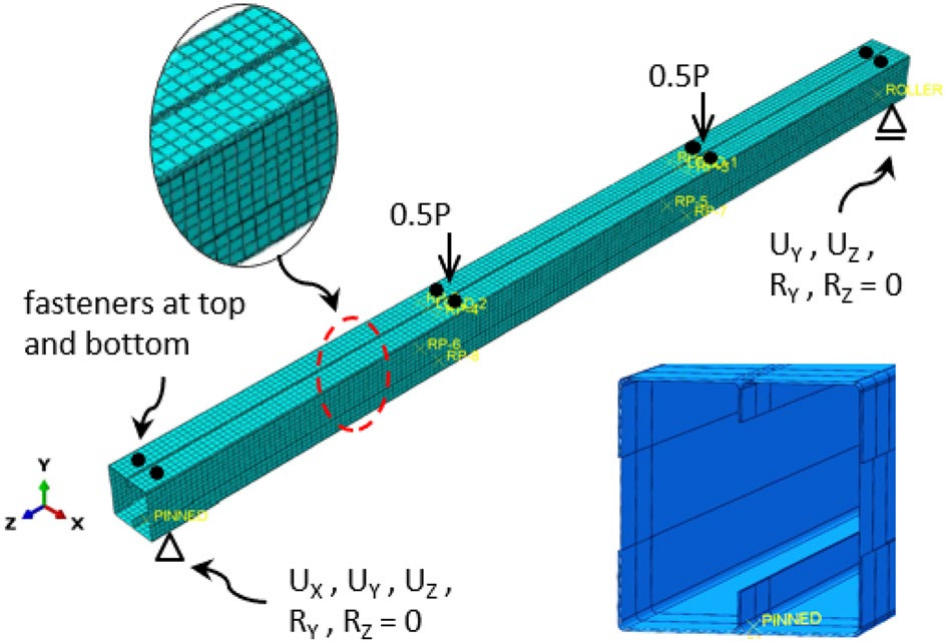
Finite element model.

Finite element analysis is conducted on the same thirteen (13) models using ABAQUS software to validate the experimental findings. A four-node thin shell element with reduced integration, denoted as S4R, is used to model the elements with a mesh size averaging 10 mm in width. The finite element model (FEM) is presented in Fig. 24 for reference. A surface-to-surface contact element incorporating a contact pressure-overclosure model in the normal direction and a frictionless model in the tangential direction is implemented for modeling contact between components.

**Figure 25:**
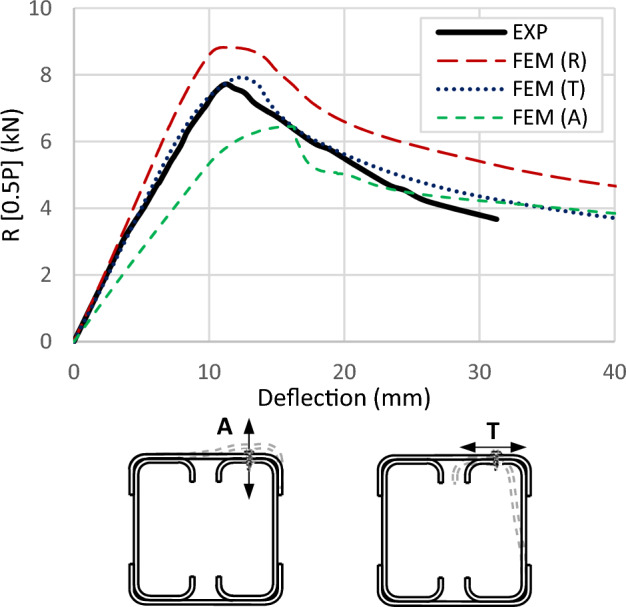
FEM for specimen S01 (92 × 92-1/1-0-G1) with different connector modules to model the screws.

A specific contact element (constraint point-based as defined in the software) is utilized in the FEM to simulate the interaction between nodes representing screws. Since these screws lack washers and nuts on the inner side, slight translations or rotations may occur. Therefore, different fasteners in the software are examined to identify the most representative one concerning the experimental outcomes. One type is a rigid connector, denoted as (R), which prevents any differential translation and rotation between the two connected nodes. Another type allows differential translation in the axial direction of the screw, denoted as (A). This type enables differential rotation in all directions; thus, it is called in the software as a slot-rotation connector. The last type is similar to the previous one but enables differential translation in the transverse direction of the screw instead of its axial direction. This type is denoted as (T). As illustrated in Fig. 25 for specimen S01 (92 × 92-1/1-0-G1), the model utilizing rigid connectors (R) demonstrates over capacity and stiffness. However, releasing translation along the screw axis (A) results in low capacity and stiffness. The model releasing one transverse translation (T) yields results most closely aligned with the experimental data. Consequently, this fastener module is adopted in the FEM as a simplified approach instead of complexly modeling the screws, threads, and sheet holes.

**Figure 26:**
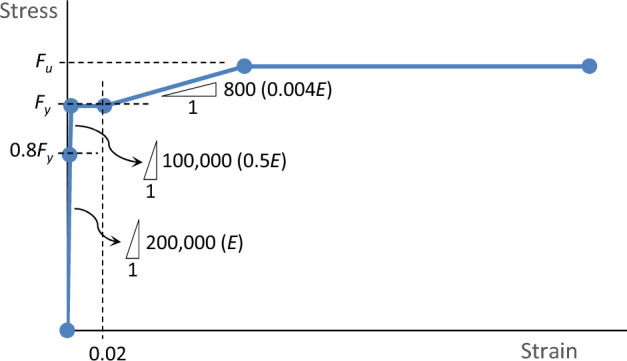
Simplified stress–strain curve in FEM.

To model the steel, isotropic von Mises material is applied. The stress–strain behavior is represented by a simplified multilinear curve derived from experimental coupon data, as shown in Fig. 26. Initially, the modulus of elasticity (*E*) is set to 200 GPa up to 80% of the yield stress. Beyond this point, a slope of 0.5*E* is used until the yield stress for relaxed elasticity. Subsequently, the curve maintains a yield plateau until a strain of 0.02, after which strain hardening occurs with a slope of 0.004*E* up to the ultimate strength. The curve then extends horizontally until failure. The main parameters of yield, ultimate stresses, and strain at fracture are provided in Table 2.

The load within FEM is simulated as an incremental displacement approach, utilizing displacement control techniques. The displacement control is the analysis method applied by activating the general-static (*STATIC) method. The non-linear geometric parameter (*NLGEOM) is also used in the FE model to account for large displacements during the analysis. The determination of the ultimate moment capacity from FEM (*M*_*FEM*_) involves multiplying reactions by their distance to the load. For boundary conditions, supports are hinged on one side and roller on the other, positioned along a line at the lower flange. A rigid body constraint is applied to the support and load locations to ensure steady stress distribution, spanning the entire surface rather than concentrating at specific nodes. Non-linear analysis is proceeded to predict the ultimate moment capacity.

**Figure 27:**
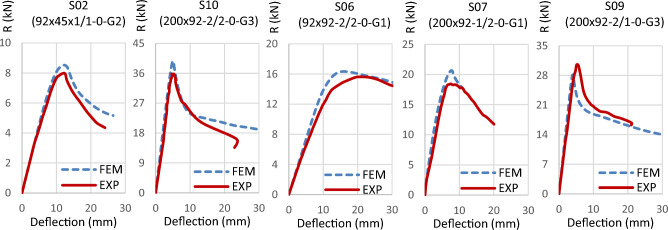
Reaction-deflection curves from FEM and experiments for different models.

**Figure 28:**
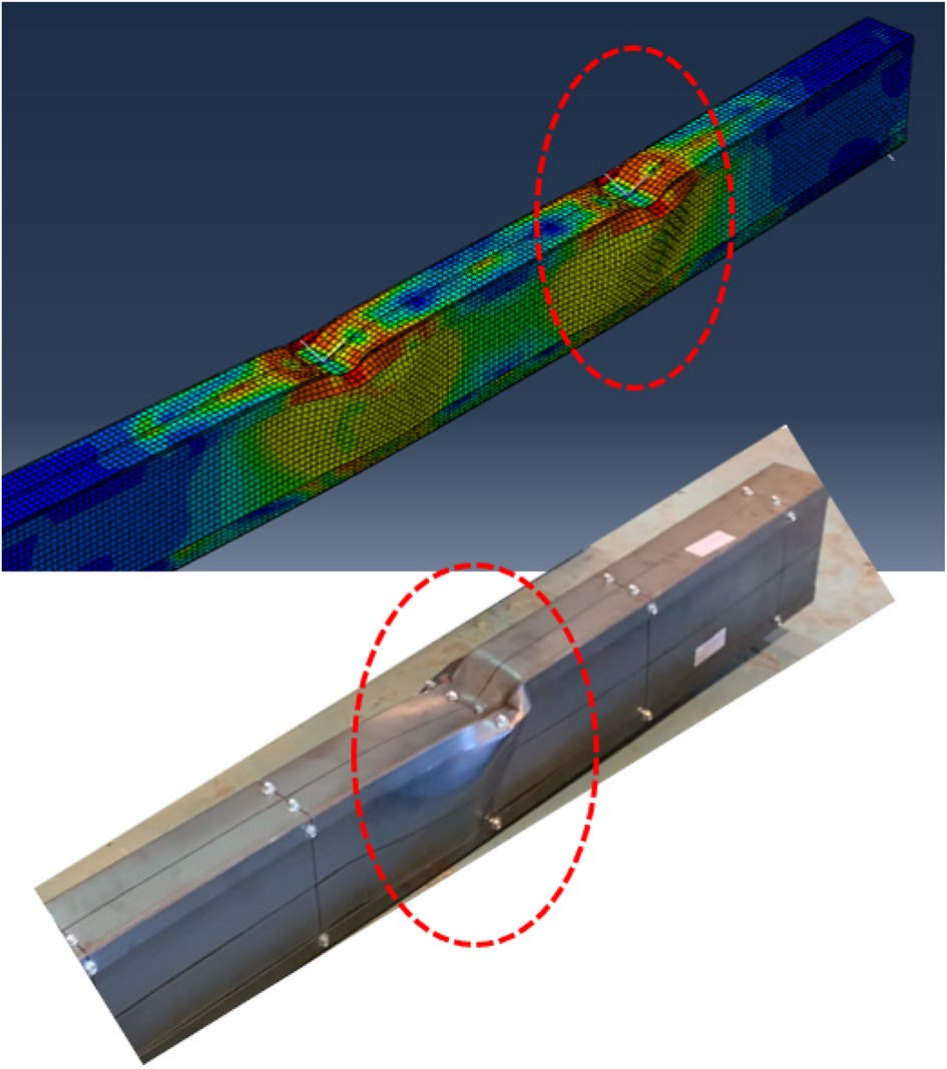
Failure mode from FEM and experiment for specimen S09 (200 × 92-2/1-0-G3).

**Figure 29:**
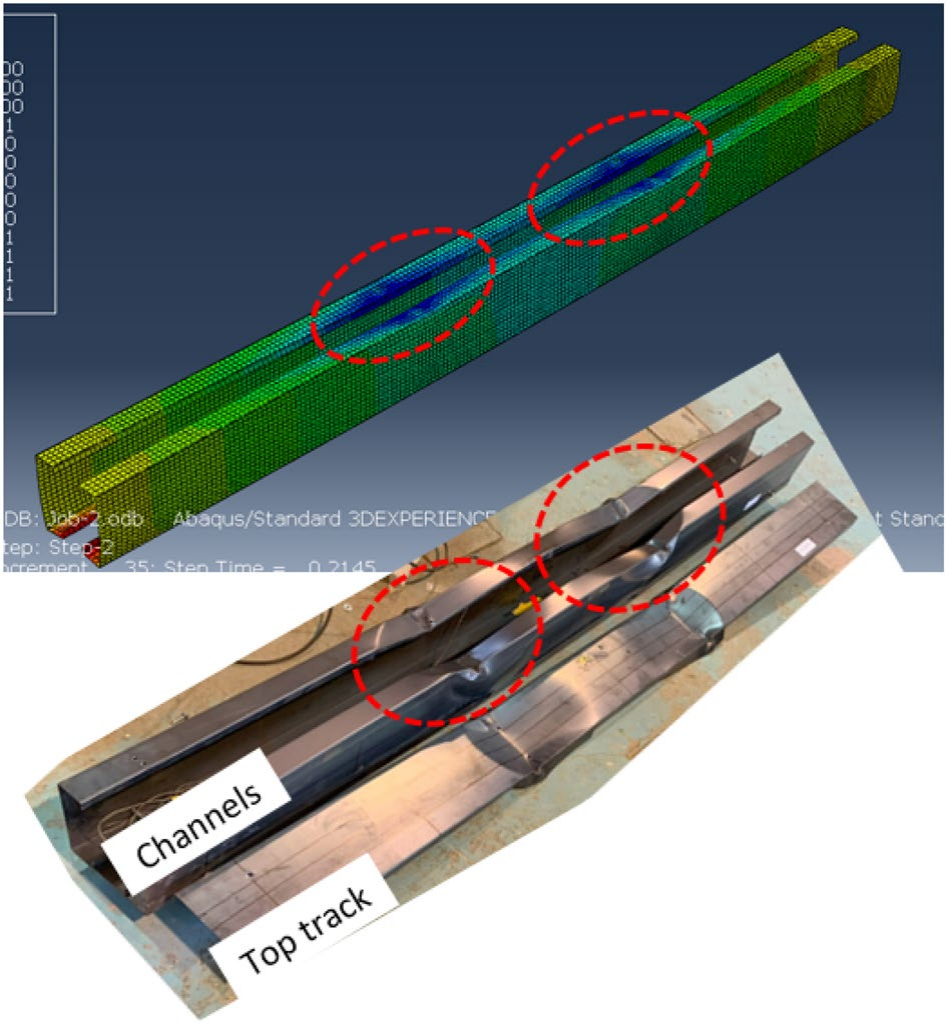
Vertical deformation of channel’s flange from FEM and experiment for specimen S11 (150 × 150-1/1-60-G1).

Validation of the FEM's accuracy is accomplished by comparing results with those from the experimental program. Table 4 summarizes this comparison, indicating a favorable correlation between FEM and experimental outcomes, with an average variation near unity (1.042) and a standard deviation of 0.095. Further verification is conducted by presenting the deformation curves in Fig. 27 and the failure modes in Figs. 28 and 29. These figures illustrate close alignment between FEM and experimental data, with discrepancies primarily emerging post-ultimate load. Overall, the FEM effectively replicates failure modes, load–displacement relationships, and ultimate capacities observed in experimental scenarios, affirming its reliability for future investigations.

**Table 4:** Results of FEM.

Specimen		*R*_*FEM*_	*R*_*FEM*_*/R*_*EXP*_
No	Designation	(kN)	
S01	92 × 92-1/1-0-G1	15.82	1.025
S02	92 × 92-1/1-0-G2	17.04	1.068
S03	92 × 92-1/2-0-G1	21.8	1.187
S04	92 × 92-1/2-0-G2	23.48	1.213
S05	92 × 92-2/1-0-G1	25.36	0.961
S06	92 × 92-2/2-0-G1	32.68	1.046
S07	200 × 92-1/2-0-G1	41.32	1.120
S08	200 × 92-1/2-0-G3	44.26	1.028
S09	200 × 92-2/1-0-G3	56.26	0.922
S10	200 × 92-2/2-0-G3	79.36	1.110
S11	150 × 150-1/1-60-G1	21.98	0.911
S12	92 × 150-1/1-60-G1	14.06	0.978
S13	92 × 150-2/1-60-G1	26.04	0.979
	Average		1.042
	Standard deviation		0.095

### Design code

The AISI standards mentioned, including AISI S212-07^3^, AISI S230-15^2^, and AISI S240-20^4^, outline design and installation requirements for header beams, including built-up box sections. While AISI S212-07^3^ served as an initial standard, it has been superseded by more recent editions of AISI S230-15^2^ and AISI S240-20^4^. These newer standards, referencing AISI S100-16^1^, provide comprehensive design guidelines for individual components, offering equations for determining bending capacity based on various failure modes. However, it is noted that the current rules for determining the capacity of built-up box sections consider the channel sections alone, neglecting any contribution from the track. Yet, it's essential to recognize that the nominal web crippling strength is the only measure accounting for the advantage of incorporating track.

The nominal moment for each specimen, as outlined in Table 5, is derived from multiplying the nominal moment of a single channel by two, with adjustments made for web crippling only as per the code. The first nominal strength is calculated taking the minimum value of (*M*_*nl*_) considering the interaction of LTB and LB, yielding moment (*M*_*y*_), and (*M*_*nd*_) from DB. The unsupported length for different buckling modes is taken equal to the total span of 1800 mm.

**Table 5:** Comparison between experimental and FEM capacities versus AISI.

Specimen		*M*_*EXP*_	*M*_*n1st*_	*M*_*EXP*_*/M*_*n1st*_	*M*_*FEM*_*/M*_*n1st*_	*M*_*n2nd*_	*M*_*EXP*_*/M*_*n2nd*_	*M*_*FEM*_*/M*_*n2nd*_
No	Designation	(kN.m)	(kN.m)			(kN.m)		
S01	92 × 92-1/1-0-G1	4.63	3.01^L^	1.537	1.575	3.66^D^	1.264	1.295
S02	92 × 92-1/1-0-G2	4.79	3.01^L^	1.589	1.696	3.66^D^	1.306	1.395
S03	92 × 92-1/2-0-G1	5.51	3.01^L^	1.828	2.170	3.66^D^	1.504	1.785
S04	92 × 92-1/2-0-G2	5.81	3.01^L^	1.927	2.337	3.66^D^	1.585	1.922
S05	92 × 92-2/1-0-G1	7.92	5.14^L^	1.539	1.479	6.38^Y^	1.240	1.192
S06	92 × 92-2/2-0-G1	9.38	5.14^L^	1.823	1.906	6.38^Y^	1.469	1.536
S07	200 × 92-1/2-0-G1	11.06	5.12^L^	2.161	2.421	5.97^V^	1.853	2.076
S08	200 × 92-1/2-0-G3	12.92	5.12^L^	2.522	2.593	5.97^V^	2.163	2.224
S09	200 × 92-2/1-0-G3	18.30	13.68^L^	1.338	1.234	17.93^C^	1.021	0.941
S10	200 × 92-2/2-0-G3	21.45	13.68^L^	1.569	1.741	17.93^C^	1.196	1.328
S11	150 × 150-1/1-60-G1	7.24	4.51^L^	1.604	1.460	6.35^D^	1.140	1.038
S12	92 × 150-1/1-60-G1	4.31	3.01^L^	1.430	1.399	3.66^D^	1.176	1.151
S13	92 × 150-2/1-60-G1	7.98	5.14^L^	1.551	1.519	6.38^Y^	1.250	1.224
	Average			1.724	1.810		1.397	1.470

^L^Indicates lateral torsional buckling failure mode including local buckling effect.

^D^Indicates distortional failure.

^Y^Indicates yielding failure mode.

^V^Indicates shear failure mode.

^C^Indicates crippling failure mode.

Regarding the nominal moment derived from the code, it is noticed that the governing failure mode of a single channel is typically the interaction between LB and LTB. In comparison to the experimental results, the capacity of built-up box sections tends to exceed the code predictions by a significant margin, ranging from 34 to 152% concerning experimental results and 23% to 159% concerning FEM analyses. Furthermore, it is observed that the experimental failure mode, primarily DB, differs from the code's prediction of LTB. The observed trend of consistently higher capacities in experimental and FEM results compared to code predictions highlights the inadequacy of the code in accurately predicting failure modes. To further explain this point, it is required to expand the comparison by including the second failure mode predicted by the code and put it together against experimental and FEM results.

As shown in Table 5, the second failure mode reference to AISI is found to be DB for six specimens, shear for two, crippling for two, and yielding for three specimens. For the six specimens of DB 2nd mode, both experimental and FEM results exhibit higher capacities by margins ranging from 14 to 59% and 4% to 92%, respectively. This increase is revealed in models including thicker tracks. While the governing code does not directly correlate with track thickness, both experimental data and FEM simulations demonstrate a positive correlation between track thickness and capacity.

The four models of 200 mm channel depth have by code high DB strength, so shear failure governs at a channel thickness of 1.0 mm, while crippling becomes dominant at 1.85 mm thickness. The shear capacity exhibits a remarkable difference from the code, often doubling the specified value evidenced by both experimental and FEM results. Conversely, in the case of crippling, specific models closely align with the code, with differences as minimal as 2% to 19%. The last three models have the second dominant mode as yielding, which still show higher capacity than code ranging from 19 to 54% in both experimental and FEM analyses.

In summary, while the AISI standards provide necessary guidelines for header beam design and installation, discrepancies exist between code predictions and experimental/FEM results, particularly regarding the capacity of built-up box sections. These differences emphasize the importance of further research and potentially revising the design standards to reflect actual performance.

### Proposal to include tracks in the flexural capacity of the built-up box section

Based on the previous analysis, it is evident that the presence of tracks significantly influences determining the flexural capacity of the built-up box section. Combining individual channels with tracks eliminates the occurrence of LTB. Consequently, relying solely on the code-specified LTB capacity of individual channels results in a conservative design. Regarding the LB of the channel flange, part of the top compression stresses transfers to the track delaying the appearance of the local buckling in the channel. Therefore, a superposition method to incorporate the track section into flexural capacity calculations is deserved. The track contribution becomes evident as experimental results demonstrate increased capacity with thicker tracks, whereas the code prescribes a constant capacity regardless of track thickness.

**Figure 30:**
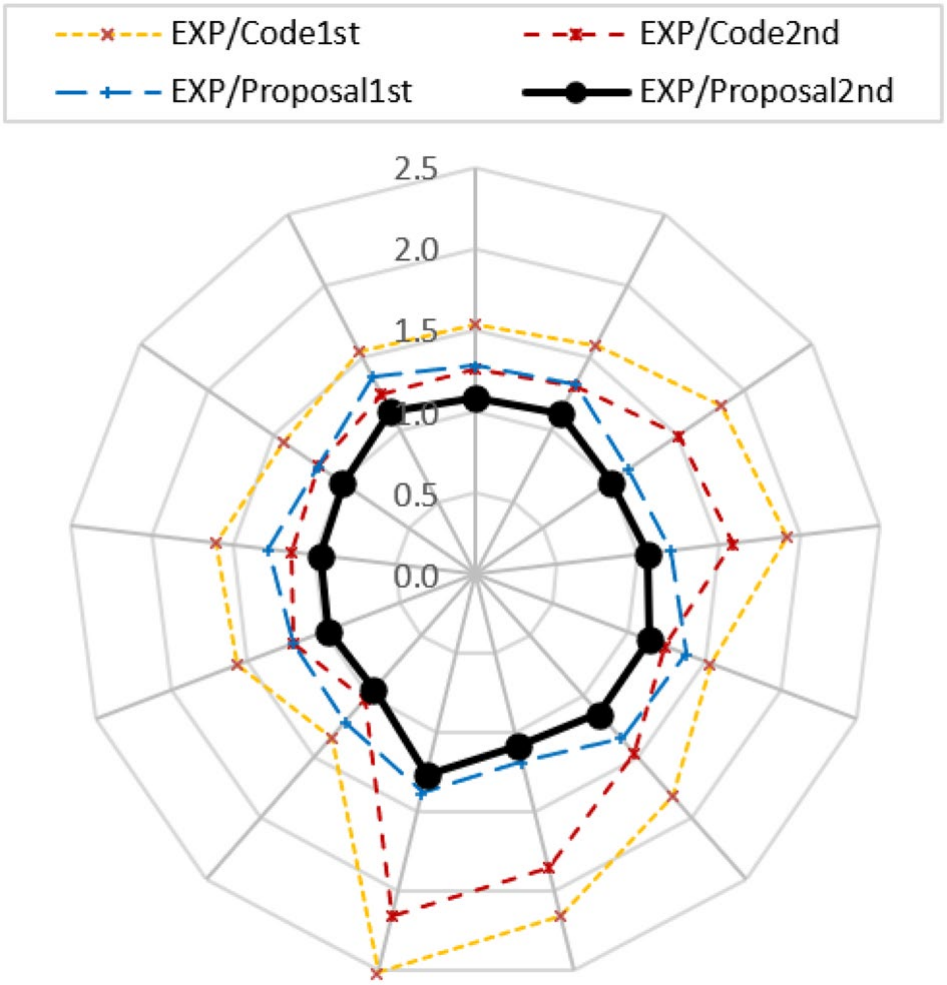
Proposal design method.

In Table 5, the code shows a conservative tendency with an average ratio of 72% compared to experimental results. Even if LTB is excluded and the second failure mode is considered the control mode, the code is still conservative by an average ratio of 39%. A proposal is made to incorporate track strength into calculations and address the code conservatism. A simple method is suggested by calculating the axial capacity of the U-section as an individual compression element and multiplying it by the arm between the center of gravity of the top and bottom tracks (Fig. 1). The nominal compression of the track is determined according to AISI^1^, considering the lowest strength from various buckling modes of the U-section. A superposition between the nominal capacity of the channels, either first or second failure mode, plus the moment from the axial force in the tracks is calculated, and then compared to experimental results and presented in Fig. 30. The figure shows that the normalized moment (*M*_*EXP*_*/M*_*proposal 1st mode*_) has a ratio in the range of 1.20. The results become closer to the unity, giving a reliable calculation than considering the individual channels only. By considering the 2nd failure mode of the channels, the normalized moment (*M*_*EXP*_*/M*_*proposal 2nd mode*_) gives values approximate to 1.0. Both methods show steady ratios for all specimens, as shown in Fig. 30. A circular arrangement for the thirteen specimens indicates trust in the results.

## Conclusion

In this study, the bending capacity of a built-up box steel section comprising two channels and two tracks is investigated, utilizing both experimental and FEM approaches. The findings offer valuable insights as follows:

- Composite action between tracks and channels is detected, resulting in an overall flexural capacity higher than the sum of the individual channel capacities.
- Tracks significantly contribute to the section's bending capacity, sometimes reaching up to 50% of the channel strains. This contribution notably increases with thicker tracks and enhances the initial stiffness and ductility.
- The slenderness of both channels and tracks influences the sharing mechanism. More slender tracks limit enhancement, while gapless channels with smaller depths exhibit the best behavior.
- Track flanges show no contribution to bending capacity. Therefore, optimizing flange width to a minimum is viable, particularly when horizontal screws are not utilized.
- In cases where track thickness exceeds that of channels, it's crucial to calculate channel bearing capacity at screws, ensuring it exceeds shear flow between track and channel.
- Adjusting screw spacing or using larger diameters becomes necessary with thicker tracks. While the close distribution of self-drilling screws showed marginal improvement, incorporating side screws into channel webs notably enhanced performance.

It is clear that the tracks, which already exist in the header beam and do not add new cost, impact the overall flexural capacity and provide optimum design beyond a 34% to 152% conservatism of the code. As a simple proposal for calculating the capacity, the compression capacity of the two tracks is to be calculated and then multiplied by the arm to the center of the built-up section. The result is to be added to the flexural capacity of the double channels to give the flexural capacity of the entire built-up section. This proposal is reliable for designing the header beams, giving a good expectation of the flexural capacity of the built-up box section with an extra safety margin of approximately 20%. Consequently, it merits inclusion in code provisions for calculating bending capacity, offering opportunities for substantial optimization in area usage or accommodating larger spans.

## Data Availability

The datasets used and/or analysed during the current study are available from the corresponding author upon reasonable request.

## Abbreviations

AISI: American iron and steel institute
C.G.: Center of gravity
DB: Distortional buckling
FEM: Finite element model
LB: Local buckling
LTB: Lateral torsional buckling
LVDT: Linear variable displacement transducers

## List of symbols

*B or D*: Total width, or depth of built-up box section; respectively [mm]
*B*_*C*_ or *B*_*T*_: Flange total width of channel, or track; respectively [mm]
*D*_*C*_ or *D*_*T*_: Section total depth of channel, or track; respectively [mm]
*E*: Modulus of elasticity of steel [MPa]
*F*_*u*_ or *F*_*y*_: Ultimate strength, or yield stress; respectively [MPa]
*P*_*EXP*_ or *P*_*FEM*_: Load from experimental tests, or finite element model; respectively [kN]
*R*_*EXP*_ or *R*_*FEM*_: Reaction from experimental tests, or finite element model; respectively [kN]
*R*_*y*_: Reaction causing yielding moment of double channels [kN]
*M*: Bending moment about the major axis (x-x) of the built-up box section [kN.m]
*M*_*EXP*_: Maximum bending moment from experimental tests [kN.m]
*M*_*FEM*_: Maximum bending moment from finite element analysis [kN.m]
*M*_*n1st*_: 1st nominal moment as per AISI due to interaction between local buckling (LB) and global buckling (LTB or Y) failure modes [kN.m]
*M*_*n2nd*_: 2nd least nominal moment as per AISI from different failure buckling modes of shear (V), crippling (C), yielding (Y), or distortional buckling (DB) [kN.m]
*P*_*EXP*_: Maximum load from experimental tests [kN]
*P*_*FEM*_: Maximum load from finite element analysis [kN]
*a*: Spacing between self-drilling screws [mm]
*b*_*C*_ or *b*_*T*_: Flange flat width of channel, or track; respectively [mm]
*d*_*C*_ or *d*_*T*_: Web flat width of channel, or track; respectively [mm]
*g*: Gap between toe-to-toe channels [mm]
*t*: Thickness (mm)
*l*_*C*_: Total depth of channel’s lip [mm]
*t*_*C*_ or *t*_*T*_: Thickness of channel, or track; respectively [mm]
*ε*_*u*_: Ultimate strain at fracture [mm/mm
